# From plate to profile: investigating the influence of dietary habits and inactive lifestyle on lipid profile in medical students at clerkship

**DOI:** 10.1186/s40795-024-00871-9

**Published:** 2024-05-07

**Authors:** Nazish Haider, Uzair Abbas, Hibba Erum Arif, Arsalan Ahmed Uqaili, Mohiba Ali Khowaja, Niaz Hussain, Mahtab Khan

**Affiliations:** 1 Dow University of Health Sciences, Karachi, Pakistan; 2 Aga Khan University, Karachi, Pakistan; 3 Liaquat University of Medical and Health Sciences, Jamshoro, Pakistan

**Keywords:** Medical students, Lipid profile, Dietary habits, Lifestyle

## Abstract

**Background:**

Dietary habits have a strong association with body lipid levels and hyperlipidemia increases the risk of cardiovascular and metabolic diseases. Dietary habits have been a major concern among medical students due to busy schedules and demanding tasks. This study was designed to know the dietary habits and lifestyle of medical students and its association with their lipid profile.

**Methods:**

We recruited 120 medical students at clerkship of the age of 18 and above. Weekly dietary habits were evaluated by an 18-item questionnaire. Five ml blood was drawn from the students and lipid profiles were measured at Dow Diagnostic Research and Reference Laboratory (DDRRL). Data was analyzed by SPSS V.22.

**Results:**

We found 70% of students were not involved in any physical activity throughout the week. Only 15.83% were following a regular diet plan. 65% of students were eating junk food for more than 3 days a week in their weekly diet. Moreover, 19.2%, 39.2%, 32.5%, and 25.84% of students were having their total cholesterol, triglycerides, HDL, and LDL levels above the optimum ranges respectively which were frequently found in students of final year (*p* < 0.05). There was high total cholesterol and LDL in males as compared to females (p value < 0.05). Total cholesterol and LDL were associated with skipped meal, use of junk food and carbonated drinks for more than 3 days a week (*p* < 0.05).

**Conclusion:**

There was a notable number of students with poor dietary habits, inactive lifestyle and lipid levels above the optimum ranges defined by American Heart Association (AHA) that have an association with dietary habits. This is alarming and can impact the health of future healthcare workers. There is a need to investigate the factors and remedies to help medical students to follow a healthy diet and a healthy lifestyle.

**Supplementary Information:**

The online version contains supplementary material available at 10.1186/s40795-024-00871-9.

## Background

Hyperlipidemia is considered as increased total cholesterol (TC) levels while dyslipidemia is imbalanced levels of Low-Density Lipoprotein (LDL), Triglycerides (TG), Very Low Density Lipoproteins (v-LDL) and High-Density Lipoprotein (HDL) levels in blood [1]. Dyslipidemia and hyperlipidemia are considered as major public health concerns as they are leading causes of vascular diseases [2]. Obesity, insulin resistance, sedentary lifestyles and high-fat diets consumption play a major role in pathophysiology of dyslipidemia [3]. According to the World Health Organization (WHO), dyslipidemia is responsible for about 2.6 million fatalities annually, with a global prevalence of 37% in males and 40% in females [4]. Youngsters of Asian subcontinent are expected to experience a significant rise in coronary artery disease incidence over the next two decades due to dyslipidemia [5]. However, healthy behaviors such as not smoking, maintaining a healthy weight, routine physical activity, and regular sleep can improve health and also prevent the development of dyslipidemia. Hence, these behaviours can prevent 80–90% of cardiovascular disease, stroke, and type 2 diabetes mellitus [6]. Preventing cardiovascular disease (CVD) requires early screening for dyslipidemia and developing effective management strategies. A 10% drop in blood cholesterol can reduce the risk of ischemic heart disease by 50% over five years [7].

College or university is a critical period during which unhealthy changes in eating behaviors and lifestyle occurs in students due to multiple reasons [8] which influence students health status. Emerging adulthood (18 to 25 years of age) is a critical time during which young people establish independence and adopt lasting health behavior patterns and it is the time associated with unhealthy lifestyle characteristics, increase risk of obesity and chronic diseases [9, 10]. Getting into medical school has an impact on a student’s health and quality of life because it requires adaptation and lifestyle changes [11]. Most medical students due to the demands of their studies and clinical rotations in the respective wards, do not get enough time to exercise and eat healthier meals [12], also the stress of university life and medical study load would be factors that negatively influence their diet [13]. The medical students are considered to have a greater knowledge about healthy lifestyle and dietary habits when compared to nonmedical students, but there is no evidence to indicate that this knowledge translates into practices in terms of maintaining good health [14]. Healthy dietary habits among medical students are very important as they are future physicians and the students who personally ignore adopting healthy lifestyle are more likely to fail to establish health promotion for their patients [12]. Early sensitization can encourage healthy lifestyle choices to maintain their health and advocate the same in their communities [15].

A cross-sectional study among medical students in Faisalabad Pakistan revealed high prevalence of unhealthy eating habits and sedentary lifestyle choices which were observed more in females [16]. An other study reported unhealthy eating habits and sedentary lifestyle with raised BMI in more than 30% of medical students affecting their academic performance also [17]. A study from Saudi Arabia reported a majority of undergraduate medical students had unhealthy eating patterns. Stress of university life and study, and lack of time for self were significantly affecting eating patterns [18]. There are multiple studies which have reported knowledge, attitude and practice of eating habits and lifestyle in medical students but there are few which have reported its association with lipid levels in those students.

Cuisine nowadays is known for its high calorie content and along with that younger generation is more likely to consume industrially processed, fast food, junk food, and sugary drinks [19, 20]. How far this consumption impacts the lipid levels in youngsters, specially in medical students has not been studied frequently. The aim of the study was to investigate the dietary habits of medical students at Dow International Medical college Karachi Pakistan. We have also evaluated the association of dietary habits and lifestyle with their lipid profile levels.

## Methodology

### Study design and setting

This cross-sectional study was conducted at Dow University of Health Sciences (DUHS) Karachi Pakistan between the period of January 2023 to November 2023 following ethical approval from the Institutional Review Board (IRB) of the university.

### Study participants

The target participants consisted of medical students at Dow International Medical College Karachi Pakistan included after a written informed consent. Students on clinical rotation of MBBS year 3, 4 and 5 were included in the study.

### Sample size calculation

A non-probability purposive sampling technique was employed to select participants [21]. The sample size was calculated from Open Epi. Knowing total number of medical students in our college as 800 and 10% estimated frequency of hyperlipidemia in our population [22], the calculated sample size was 119. However, we included a total of 120 participants.

### Inclusion and exclusion criteria

In a bachelor program of 5 years, we included the students on clinical rotation in year 3–5 as they have rotational evening and night duties in hospital during clerkship. The study and procedure were explained to students in the classrooms, those who agreed to participate were included in the study. The study excluded students who were newly admitted or had not attended clinical rotation (year 1–2). We also excluded the students who did not agree to participate in the study.

### Data and sample collection

After a written informed consent, students were asked to fill in a validated questionnaire of 18 items. The questionnaire included information regarding their dietary habits and lifestyle based on their weekly routine. Afterwards, 5 ml blood was collected in laboratory under same conditions (minimum 12 h fasting) and sent for measuring lipid profile levels to Dow Diagnostic Research and Reference Laboratory (DDRRL).

### Data collection tool

A self-constructed questionnaire was developed in collaboration with the department of Nutritional Health. To safeguard anonymity, the identities of the participants were held in strict confidence. The questionnaire was piloted on 5 participants out of the study participants and modifications were made after suggestions. The final questionnaire was validated by the Department of Medical Education of our university and approved by the Institutional Review Board. (Supplementary file S1).

The data collection tool was comprised of a total of 18 items divided into their (i) personal and demographic information, (ii) weekly dietary habits and (iii) lifestyle (exercise habits, involvement in sports) for days per week. Further we divided the response of “days per week” into none or occasional, ≤3 days a week and >3 days a week. Serum lipid profile ranges were set and compared as per guidelines of American Heart Association [23]. The optimum ranges of total cholesterol levels were set as < 200 mg/dl; Triglycerides ≤ 150 mg/dl; LDL < 100 mg/dl; HDL > 40 mg/dl for males and < 50 mg/dl for female.

### Data analysis

The data obtained from the respondents were analyzed using Microsoft Excel. Frequencies and percentages were presented in tabulated form. The analysis was performed on SPSS version 22.0. T test was performed to compare the means of continuous variables however, Pearson’s chi square was used to compare the categorical data.

## Results

### Demographic characteristics of students

Forty students were included from each year (3rd , 4th and 5th year). The mean age of students was 20.95 ± 2.05 years. There was equal number of males and females in the study. Mean BMI of the students was 21.89 ± 3.95 Kg/m^2^. Twenty-three (23%) of students had a family history of diabetes. Hypertension, hyperlipidemia, or cardiovascular disorders.

### Lifestyle of medical students

We found 84/120 (70%) students were not exercising in routine while 27/36 (75%) exercisers were limited to either walking or running and only 3/36 (8.33%) were doing aerobic exercises in their weekly routine. Most of them 23/36 (63.88%) had ≤ 3 days of exercise included in their weekly routine and 84/120 (70%) were not participating in any sports activity in a week (Table 1).

**Table 1:** Lifestyle of medical students *n* = 120

Variables	Total	Response	Frequency	Percentage
Exercise / physical activity	120	Yes	36	30
		No	84	70
Type of exercise / physical activity	36	Aerobics	3	8.33
		gym	4	11.11
		Walk/running	27	75
		Yoga	2	5.55
Exercise / physical activity days per week	36	≤ 3days	23	63.88
		> 3 days	13	36.11
Participation in sports	120	Yes	46	38.33
		No	74	61.66
Type of Sports	46	Indoor	27	58.69
		Outdoor	19	41.30

The table shows the routine of physical activity, participation in sports and type of sports and physical activity performed by the students in a week

### Dietary habits of students

We observed 101/120 (84.16%) students were not following a regular diet plan and 53.33% were found to skip their breakfast on usual days. Moreover, 116/120 (96.66%) students reported eating vegetables in their weekly diet for less than 3 days of week and 53/120 (44.16%) used to eat meat for more than 3 days in a week. Most of the students (94.16%) eat fruit only for less than 3 days a week. A large number (78/120) of students eat junk food for more than 3 days a week. We also found that 67.5% of students were using carbonated drinks for at least 3 days a week (Table 2).

**Table 2:** Dietary habits of students *n* = 120

Variables	Response	Frequency n	Percentage %
Following a regular diet plan	Yes	19	15.83
	No	101	84.16
Most skipped meal of the day	Breakfast	64	53.33
	Lunch	26	21.66
	Dinner	8	6.66
	None	22	18.33
Use of vegetables	None/occasional	02	1.66
	≤ 3days	116	96.66
	> 3 days	02	1.66
Use of meat	None/occasional	00	-
	≤ 3days	53	44.16
	> 3 days	67	55.83
Use of fruits	None/occasional	02	1.66
	≤ 3days	113	94.16
	> 3 days	05	4.16
Use of dry fruits	None/occasional	98	81.66
	≤ 3days	14	11.66
	> 3 days	08	6.66
Use of junk foods	None/occasional	02	1.66
	≤ 3days	40	33.33
	> 3 days	78	65
Use of energy drinks	None/occasional	56	46.66
	≤ 3days	42	35
	> 3 days	22	18.33
Use of carbonated drinks	None/occasional	11	9.16
	≤ 3days	81	67.5
	> 3 days	28	23.33

The table shows the number and percentage of dietary habits of medical students. Responses were collected as non or occasional, ≤ 3 days or  >3 days per week

### Difference in dietary habits of students according to their year of study

Students based on their year of study, the least number of students who were following a regular diet plan were from year 5 with a significant difference as compared to year 3 and year 4 (*p* = 0.001). Most of the students who skipped their breakfast usually, were also from the year 5 (*p* < 0.001). Most of the junk food eaters for more than 3 days were also found in year 5 (*p* < 0.001). However, 31/81 students who used to drink carbonated drinks were from year 3 (*p* = 0.024; Table 3).

**Table 3:** Differential dietary habits of medical students according to their year of study *N* = 120

Variables	Response	*N* = 120	Year 3 *N* = 40	Year 4 *N* = 40	Year 5 *N* = 40	*P* value
		n	n/%	n/%	n/%	
Following a regular diet plan	Yes	19	13 (32.5)	04 (10)	02 (5)	0.001
	No	101	27 (67.5)	36 (90)	38 (95)	
Most skipped meal of the day	Breakfast	64	13 (32.5)	19 (47.5)	32 (80)	< 0.001
	Lunch	26	09 (22.5)	11 (27.5)	06 (15)	
	Dinner	8	04 (10)	03 (7.5)	01 (2.5)	
	None	22	14 (35)	07 (17.5)	01 (2.5)	
Use of vegetables	None/occasional	02	02 (5)	-	-	0.012
	≤ 3days	116	36 (90)	40 (100)	40 (100)	
	> 3 days	2	02 (5)	-	-	
Use of meat	None/occasional	00	-	-	-	< 0.001
	≤ 3days	53	13 (32.5)	22 (55)	18 (45)	
	> 3 days	67	27 (67.5)	18 (45)	22 (55)	
Use of fruits	None/occasional	02	02 (5)	-	-	0.51
	≤ 3days	113	35 (87.5)	39 (97.5)	39 (97.5)	
	> 3 days	5	03 (7.5)	01 (2.5)	01 (2.5)	
Use of dry fruits	None/occasional	98	29 (72.5)	35 (87.5)	34 (85)	0.004
	≤ 3days	14	06 (15)	03 (7.5)	05 (12.5)	
	> 3 days	08	05 (12.5)	02 (5)	01 (2.5)	
Use of junk foods	None/occasional	2	0	01 (2.5)	01 (2.5)	< 0.001
	≤ 3days	40	24 (60)	16 (40)	06 (15)	
	> 3 days	78	16 (40)	23 (57.5)	33 (82.5)	
Use of energy drinks	None/occasional	56	19 (47.5)	14 (35)	23 (57.5)	0.517
	≤ 3days	42	10 (25)	18 (45)	14 (35)	
	> 3 days	22	11 (27.5)	08 (20)	03 (7.5)	
Use of carbonated drinks	None/occasional	11	03 (7.5)	06 (15)	02 (5)	0.024
	≤ 3days	81	34 (85)	22 (55)	25 (62.5)	
	> 3 days	28	03 (7.5)	12 (30)	13 (32.5)	

The table shows the number and percentage of dietary habits of medical students Responses were collected as non or occasional, ≤ 3days or >3 days per week. Pearson’s Chi square test was used to see the differential dietary habits among year 3, 4 and 5. P value less than 0.05 was considered as significant at 95% CI

### Lipid profile of students

**Fig. 1:**
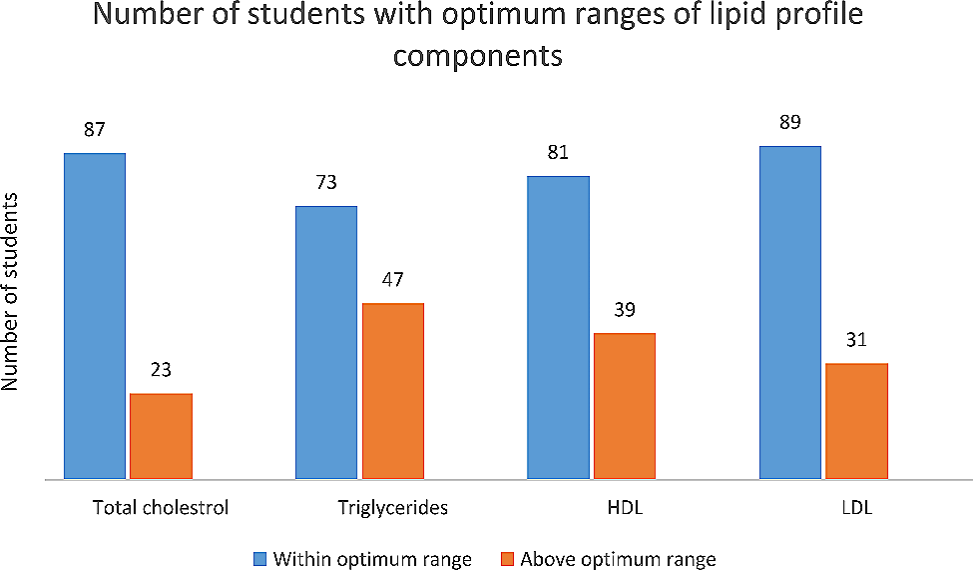
Lipid profile of students The figure shows number of students with optimum ranges of lipid profile components as per American Heart Association (AHA). The optimum ranges of total cholesterol were set as < 200 mg/dl; Triglycerides ≤ 150 mg/dl; LDL < 100 mg/dl; HDL < 40 mg/dl for males and < 50 mg/dl for female. *n* = 120

We divided the ranges of cholesterol levels according to AHA guidelines. We found 19.2% of students had total cholesterol levels above the optimum ranges. For the triglycerides, HDL, and LDL, 47/120 (39.2%), 39/120 (32.5%) and 31/120 (25.84%) students were found to fall in above optimum ranges of lipid levels respectively (Fig. 1).

### Lipid profile of the students based on gender

**Fig. 2:**
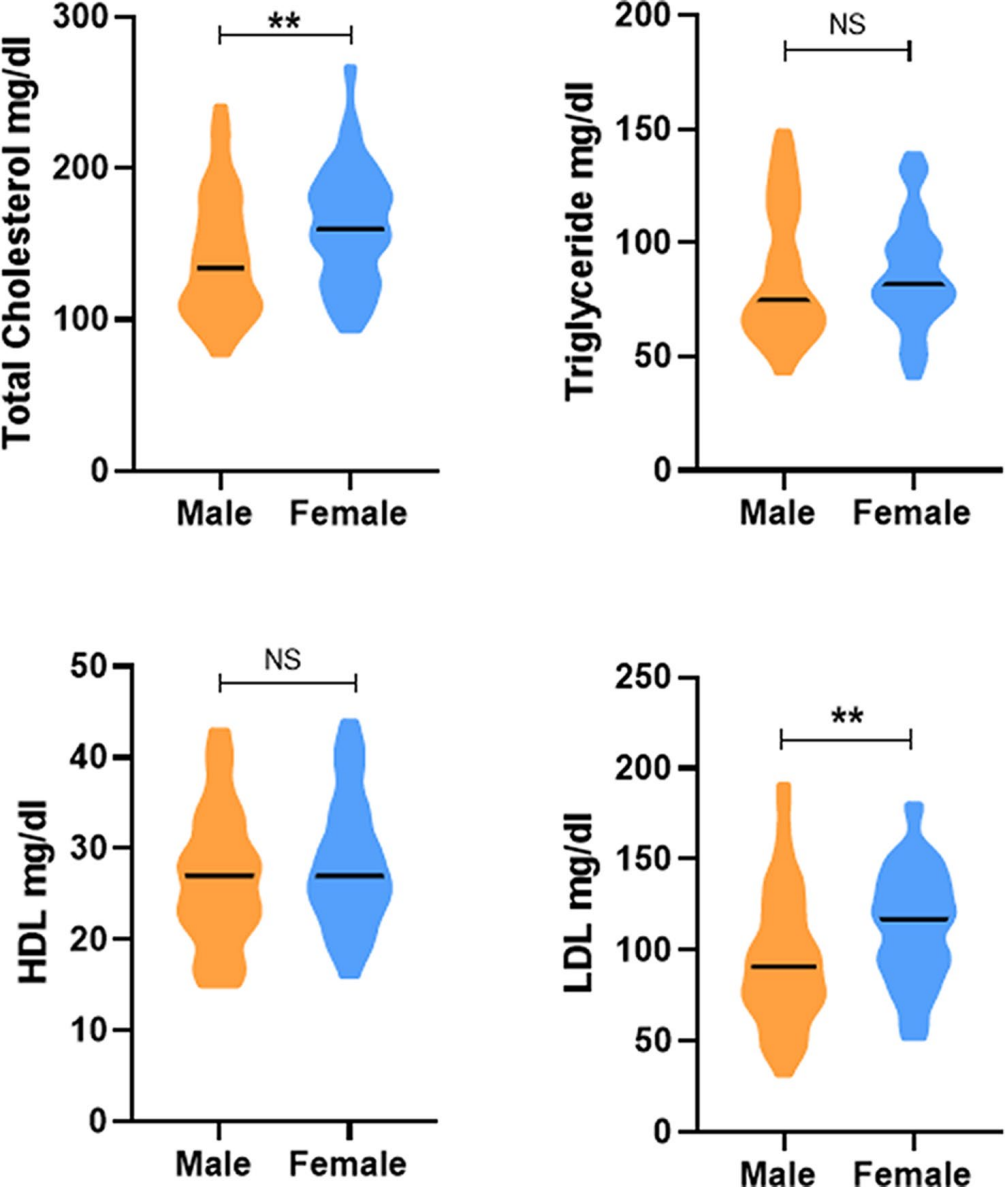
Differential lipid profile of medical students based on gender. The Violin graphs show median lipid levels in mg/dl among male and female students. **A**: total cholesterol **B**: Triglycerides **C**: HDL **D**: LDL; p value less than 0.05 was considered as significant

We found a higher total cholesterol level in males as compared to females (median 160.0 vs. 134.0; p value = 0.0034). We also found high levels of LDL in males as compared to females (117.0 vs. 91.0; p value = 0.0015) but no difference was found in triglyceride and HDL levels between male and females (Fig. 2).

### Differential lipid profile of students according to their year of study

We found a higher number 12/40 (30%) of students of year 5 had cholesterol levels > 200 mg/dl but no significant difference between year of study was found(*p* = 0.117) however, those who had triglycerides > 150 mg/dl were from year 5 with a significant difference from year 3 and 4 (*p* = 0.003). Moreover, students with LDL more than 100 mg/dl and HDL less than 40 mg/dl were also from year 5 with a significant difference (*p* = 0.047 and 0.024 respectively; Table 4).

**Table 4:** Differential lipid profile of students according to their year of study *n* = 120

Lipid profile Variables	Ranges (mg/dl)	*N* = 120	Year 3 *N* = 40	Year 4 *N* = 40	Year 5 *N* = 40	P value
		n/%	n/%	n/%	n/%	
Total cholesterol	≤ 200	97 (80.8)	36 (90)	33 (82.5)	28 (70)	0.117
	> 200	23 (19.2)	4 (10)	7 (17.5)	12 (30)	
Triglycerides	≤ 150	73 (60.8)	31 (87.5)	24 (60)	18 (45)	0.003*
	> 150	47 (39.2)	9 (22.5)	16 (40)	22 (55)	
LDL	< 100	89 (74.16)	30 (75)	33 (82.5)	26 (65)	0.047*
	≥ 100	31 (25.84)	10 (25)	7 (17.5)	14 (35)	
HDL	> 40	81 (67.5)	33 (82.5)	29 (72.5)	19 (47.5)	0.024*
	≤ 40	39 (32.5)	7 (17.5)	11 (27.5)	21 (52.5)	

The table shows differential lipid profile (categorized in below optimum and above optimum ranges) according to year of study. Pearson’s Chi square test was used to see the differential lipid profile of students studying in year 3, 4 and 5. P value less than 0.05 was considered as significant at 95% CI

### Association of lipid profile with dietary habits

We further analyzed if there is an association of lipid profile with dietary habits. We found that total cholesterol levels were associated with skipped meals and use of junk food for more than 3 days a week (p value 0.003 and 0.02 respectively). We also found an association of LDL with usage of carbonated drinks and physical activity for less than 3 days a week (*p* = 0.002 and 0.043 respectively). Moreover, we did not find any association of HDL and triglyceride levels with any study variables.

## Discussion

The findings of our study offer valuable insights of lifestyle, dietary habits on a weekly basis, and their association with lipid profiles in medical students. We observed 70% of students maintaining a sedentary lifestyle and 84% were unable to follow a regular diet plan which includes 70% of students skipping at least one regular meal. This was more frequently reported in students in the final year of their clerkship. We also found 19–25% of students having a lipid level above the optimum ranges as defined by AHA.

We observed 70% of students were not exercising in their weekly routine indicating a significant proportion of the participants maintaining a sedentary lifestyle, which highlights an urgent matter concerning the health and well-being of medical students. University students in Pakistan tend to be sedentary and fail to engage in regular physical exercises, according to a survey [24]. The present study’s findings that this inactive lifestyle increases the likelihood of metabolic syndrome are in line with those of the previous research in Kenya [25]. Furthermore, consistent trends were found in a research from a medical college from Pakistan, reporting a significant number of medical students with a sedentary lifestyle and only few were following a regular aerobic exercise [26], the current study found that only 3 out of 56 individuals were engaged in aerobic activity. The present study’s findings on inadequate aerobic exercise involvement are in line with the wider pattern of sedentary behavior reported among young adults in Pakistan [27] and globally [28]. This lifestyle is widely prevalent in medical students due to demanding tasks during clerkship. As the students proceed to their higher years of education in medical life, the routine becomes tough and they get lesser time for physical activities [17].

The individuals’ dietary habits were also examined, with an emphasis on the amount of trans fatty acids such as junk food they consumed in a week. We observed 84% of students were not following a regular diet plan and up to 70% were found to skip a single meal of the day. The study’s findings regarding the poor dietary practices of medical students are comparable with and supported by an abundance of prior research in Pakistan and worldwide [29]. Our findings are consistent with a prior investigation which has reported 49% and 50% of students not following a regular diet plan and skipping meals frequently [14]. A study from Saudi Arabia reported that only 37.4% of the population in their sample size consumed breakfast regularly while remaining used to skip, and two-thirds of students ate junk food more often [30]. An other study from Saudi Arabia in 2020 reported a majority of undergraduate medical students had unhealthy eating patterns, and socioeconomic and psychological elements were significantly affecting eating patterns [18]. We also found 65% of the participants consumed a considerable quantity of junk food which coincides with results from a study which also identifies high intake of junk food and these fatty acids were identified as substantial dietary risk factors for non-communicable diseases [31].

We also found 94% of students had very small portions of vegetables and fruits in their weekly routine. These results are much higher than the previously reported data from Maharashtra, India in which they showed 75% of students used to eat vegetables in a negligible portion of routine diet [32]. Researchers also found that of the students surveyed, 64.7% were not active at all, and 52.4% watched more than two hours of television every day. Fruits and vegetables were not consumed in sufficient quantities (14% and 6.8%, respectively). At least once every eleven days, 37.1% of the population ate junk food [33]. In addition, a prior study found that a quarter of students did not include fruit in their daily diet and that 21.5% of students eat snacks in addition to their normal meals [29]. The ongoing difficulty of consuming a balanced and healthy diet has been highlighted in international study [34] as has the current study. Medical students are supposed to have a better understanding of healthy eating habits, instead of this, the high prevalence of poor eating habits is alarming.

Moving ahead, we compared the dietary habits among medical students based on their year of study or clerkship. We found high consumption of carbonated drinks, junk food, least consumption of vegetables and fruits along with skipping meals in medical students of final year as compared to other level of study. Though there are few studies which have compared dietary habits with respect to year of study, a Sudanese study is consistent with the present study’s finding that a significant percentage of students, especially those in their fifth year, skip breakfast at all and use junk food frequently [35].

Furthermore, an alarming association between dietary patterns and lifestyle patterns was observed with elevated cholesterol levels in medical students who participated in this research. In line with a study in 2020, that found a high prevalence of elevated cholesterol levels among students with poor dietary habits, such as skipping meals and frequent consumption of fast food [36], the observation in our study that 19% of students had total cholesterol levels above the optimal ranges is consistent with the past literature findings. The results of our study, which revealed that for the triglycerides, HDL, and LDL, 39%, 32% and 25% of students were found to fall in above optimum ranges of lipid levels respectively support an expanding body of literature that emphasizes the correlation between unhealthy eating patterns and unfavorable lipid profiles [37]. The need to address dietary behaviors to reduce the risk of high cholesterol among students is highlighted by these consistent findings [38]. A research in China also showed that increased levels of physical activity and adherence to a dietary pattern rich in high-quality protein foods, vegetables, and fruits were found to be linked to positive lipid profiles [39]. A sedentary lifestyle and poor dietary habits or high intake of trans fatty acids, saturated fats, and refined sugars found in these dietary sources promotes dysregulation of lipid metabolism, leading to elevated LDL cholesterol levels and diminished HDL cholesterol levels. Additionally, inadequate consumption of fruits and vegetables deprives individuals of essential nutrients and antioxidants necessary for maintaining optimal lipid profiles and cardiovascular health [40].

Additionally, this research’s results that carbonated drink use is associated with elevated LDL levels and insufficient physical activity for fewer than three days per week are in line with the Framingham heart study [41]. A Nigerian study reported that carbonated soft drinks are used by 29.0% which is associated with incidence of non-communicable disease in their student population [42]. Another study in Kenya revealed that only 2.2% of the respondents were out of shape, while 61.3% did not exercise routinely and 72.3% snacked frequently. Additionally, 37.2% required to improve their fitness [25]. Nonetheless, the results of research showed no significant relationships have been observed between HDL and triglyceride levels with any study variables. These results contradict the outcome of a study that suggested plant-based diets have the potential to impact triglycerides, HDL levels, and anthropometric properties based on their composition [43].

## Conclusion

There was a notable number of students with poor dietary habits, inactive lifestyle and lipid levels above the optimum ranges defined by American Heart Association (AHA) that have an association with dietary habits. This effect was prominent among students of year 5 of their study. This is alarming and can impact the health of future healthcare workers. We recommend investigating the factors and remedies to help medical students to follow a healthy diet and a healthy lifestyle.

### Electronic supplementary material

Below is the link to the electronic supplementary material.

Supplementary Material 1

## Data Availability

All data has been included in the study however it is available with the corresponding author and may be provided on request.

## Abbreviations

TC: Total Cholesterol
LDL: Low-Density Lipoprotein
TG: Triglycerides
VLDL: Very Low Density Lipoproteins
HDL: High-Density Lipoprotein
AHA: American Heart Association
DM: Diabetes Mellitus
DDRRL: Dow Diagnostic Research and Reference Laboratory
DIMC: Dow International Medical College

## References

[1] Su X, Chen X, Wang B (2021). Pathology of metabolically-related dyslipidemia. Clin Chim Acta.

[2] Vekic J, Stefanovic A, Zeljkovic A (2023). Obesity and dyslipidemia: a review of current evidence. Curr Obes Rep.

[3] Clemente-Suárez VJ, Beltrán-Velasco AI, Redondo-Flórez L, Martín-Rodríguez A, Tornero-Aguilera JF. Global impacts of western Diet and its effects on Metabolism and Health: a narrative review. Nutrients. 2023;15. 10.3390/nu15122749. 10.3390/nu15122749 PMC10302286 37375654

[4] Ahmed B, Konje JC. The epidemiology of obesity in reproduction. Best Pract Res Clin Obstet Gynecol 2023:102342. 10.1016/j.bpobgyn.2023.102342 37276817

[5] Martinez-Amezcua P, Haque W, Khera R, Kanaya AM, Sattar N, Lam CS (2020). The upcoming epidemic of heart failure in South Asia. Circulation: Heart Fail.

[6] Kaminsky LA, German C, Imboden M, Ozemek C, Peterman JE, Brubaker PH (2022). The importance of healthy lifestyle behaviors in the prevention of cardiovascular disease. Prog Cardiovasc Dis.

[7] Thongtang N, Sukmawan R, Llanes EJB, Lee ZV (2022). Dyslipidemia management for primary prevention of cardiovascular events: best in-clinic practices. Prev Med Rep.

[8] DeBate RD, Topping M, Sargent RG (2001). Racial and gender differences in weight status and dietary practices among college students. Adolescence.

[9] Deshpande S, Basil MD, Basil DZ (2009). Factors influencing healthy eating habits among college students: an application of the health belief model. Health Mark Q.

[10] Nelson MC, Story M, Larson NI, Neumark-Sztainer D, Lytle LA (2008). Emerging adulthood and college-aged youth: an overlooked age for weight-related behavior change. Obesity.

[11] Tempski P, Bellodi PL, Paro HB, Enns SC, Martins MA, Schraiber LB (2012). What do medical students think about their quality of life? A qualitative study. BMC Med Educ.

[12] Agha SA, Agha MA, Usman G, Agha Z. Assessment of the perceptions of health among medical students. Gomal J Med Sci 2011, 9.

[13] Ganasegeran K, Al-Dubai SA, Qureshi AM, Al-Abed A-AA, Am R, Aljunid SM (2012). Social and psychological factors affecting eating habits among university students in a Malaysian medical school: a cross-sectional study. Nutr J.

[14] Sajwani RA, Shoukat S, Raza R, Shiekh MM, Rashid Q, Siddique MS (2009). Knowledge and practice of healthy lifestyle and dietary habits in medical and non-medical students of Karachi, Pakistan. J Pak Med Assoc.

[15] Sharma R, Mandliya J, Dhaneria M, Tiwari HJIJMRR. Prevalence of hypertension in mid adolescents in central India: a school based comparative study. 2015, 3:891–9.

[16] Zuhair HMU, Fatima K, Hussain U, Ayub A (2024). Assessment of eating habits, lifestyle and physical activity among medical and dental students of Faisalabad Medical University. Pakistan J Med Sci.

[17] Joshi BP, Mahajan SM, Tayade DN (2023). Physical activity and its correlation with various measures of obesity among medical students and young faculty. Clin Epidemiol Global Health.

[18] Alzahrani SH, Saeedi AA, Baamer MK, Shalabi AF, Alzahrani AM. Eating habits among medical students at king abdulaziz university, Jeddah, Saudi Arabia. Int J Gen Med 2020:77–88. 10.2147/IJGM.S246296 PMC7062392 32184649

[19] Chandio MK, Memon MSS, Hashim H, Muhammad SD, Talpur MS, Naveel TJJoPNR. PREVALENCE OF OBESITY AMONG MEDICAL STUDENTS OF PAKISTAN, AND ITS ASSOCIATION WITH PHYSICAL ACTIVITY AND DIETARY HABITS. 2022:2411–9.

[20] Tarar OM, Ahmed KM, Nishtar NA, Achakzai ABK, Gulzar Y, Delles C (2020). Understanding the complexities of prevalence of trans fat and its control in food supply in Pakistan. J Clin Hypertens (Greenwich Conn).

[21] Setia MS (2016). Methodology series module 5: sampling strategies. Indian J Dermatology.

[22] Zeeshan G, Naimat W, Furqan H, Shuja H (2023). Prevalence, risk factors and implications of hypercholesterolemia in Pakistan. IJS Global Health.

[23] Piazza G, Desai NR, Baber U, Exter J, Kalich B, Monteleone P. Practical Solutions for implementation of blood cholesterol guidelines in clinical practice. Trends Cardiovasc Med 2023. 10.1016/j.tcm.2023.08.001 37634754

[24] Ullah I, Islam MS, Ali S, Jamil H, Tahir MJ, Arsh A et al. Insufficient physical activity and sedentary behaviors among medical students during the COVID-19 lockdown: findings from a cross-sectional study in Pakistan. 2021, 18:10257. 10.3390/ijerph181910257 PMC8508425 34639559

[25] Mbugua SM, Munyoki G, Kimani STJ. The Association of Physical Activity and Diet with metabolic syndrome among University students in Kenya. 2020, 6:106–14.

[26] Gulzar S. Physical Activity Levels among Young Adolescent Students in Urban Karachi, Pakistan. 2021.

[27] Malik MS, Qayyum W, Farooq A, Waqas A, Sukhera AB, Khalid MA et al. Dietary patterns, exercise, and the metabolic syndrome among young people in Urban Pakistan (Lahore). 2020, 18:56–64. 10.1089/met.2019.0021 31638468

[28] Abd-Allatif REE. PHYSICAL ACTIVITY PREVALENCE AMONG MIGRANTS IN UNITED ARAB EMIRATE. 2019.

[29] Fauz R, Hani U, Batool S, Javaid MJTT. Awareness of Dietary Habits and Balanced Lifestyle Among Physical Therapy Students: Awareness of Dietary Habits and Balanced Lifestyle. 2023:52 – 6.

[30] Alzahrani SH, Saeedi AA, Baamer MK, Shalabi AF, Alzahrani AMJIjogm. Eating habits among medical students at king abdulaziz university, Jeddah. Saudi Arabia 2020:77–88. 10.2147/IJGM.S246296 PMC7062392 32184649

[31] Tarar OM, Ahmed KM, Nishtar NA, Achakzai AB, Gulzar Y, Delles C et al. Understanding the complexities of prevalence of trans fat and its control in food supply in Pakistan. 2020, 22:1338–46. 10.1111/jch.13943 PMC8029707 32687252

[32] Vibhute NA, Baad R, Belgaumi U, Kadashetti V, Bommanavar S, Kamate W (2018). Dietary habits amongst medical students: an institution-based study. J Family Med Prim care.

[33] Rahamathulla MP (2020). Frequency and awareness of risk factors of non-communicable diseases among University students in Saudi Arabia. Pakistan J Med Sci.

[34] Al-Awwad NJ, Al-Sayyed HF, Zeinah ZA, Tayyem RF (2021). Dietary and lifestyle habits among university students at different academic years. Clin Nutr ESPEN.

[35] Al-Haj MEA, Awooda HA, Elnimeiri MKM (2015). Eating habits among medical students in a Sudanese medical faculty. Int Res J Med Med Sci.

[36] Kolobarić N, Gradinjan Centner M, Šušnjara P, Matić A, Drenjančević IJIJER, Health P. Anthropometric and biochemical parameters in relation to dietary habits as early indicator of cardiovascular impairment in young adult cohort. 2020, 17:9208. 10.3390/ijerph17249208 PMC7764557 33317131

[37] Gomez-Delgado F, Katsiki N, Lopez-Miranda J, Perez-Martinez, PJCrifs. nutrition. Dietary habits, lipoprotein metabolism and cardiovascular disease: From individual foods to dietary patterns. 2021, 61:1651-69. 10.1080/10408398.2020.1764487 32515660

[38] Gherasim A, Arhire LI, Niță O, Popa AD, Graur M, Mihalache LJPNS (2020). Relatsh between Lifestyle Compon Diet Patterns.

[39] Guo Q, Ma Z, Zhu C, Zeng QJLiH. Disease. Association of dietary pattern and physical activity with lipid-related indices among Chinese population: a cross-sectional study. 2020, 19:1–13. 10.1186/s12944-020-01420-6 PMC7684929 33228692

[40] Dobe M (2024). Nutrition, Diet, and Health: role of macronutrients, micronutrients, and Nutraceuticals.

[41] Haslam DE, Peloso GM, Herman MA, Dupuis J, Lichtenstein AH, Smith CE et al. Beverage consumption and longitudinal changes in lipoprotein concentrations and incident dyslipidemia in US adults: the Framingham heart study. 2020, 9:e014083. 10.1161/JAHA.119.014083 PMC7335580 32098600

[42] Olatona FA, Onabanjo OO, Ugbaja RN, Nnoaham KE, Adelekan DA. Dietary habits and metabolic risk factors for non-communicable diseases in a university undergraduate population. *Journal of health, population, and nutrition* 2018, 37:21, 10.1186/s41043-018-0152-2. 10.1186/s41043-018-0152-2 PMC6097209 30115131

[43] Borazjani M, Nouri M, Venkatakrishnane K, Najafi M, Faghih SJN, Science F. Association of plant-based diets with lipid profile and anthropometric indices: a cross-sectional study. 2022, 52:830–42.

