# Supplementary material for: From plate to profile: investigating the influence of dietary habits and inactive lifestyle on lipid profile in medical students at clerkship
Source: BMC Nutr. 2024 May 7;10:71. doi: 10.1186/s40795-024-00871-9 (PMC11077723; doi:10.1186/s40795-024-00871-9)
Supplement: Supplementary file 1 — Supplementary Material 1 [file 40795_2024_871_MOESM1_ESM.pdf]

Impact of dietary habits and lifestyle on lipid profile of medical students
Data collection form

Serial no: ________ Date: _________ Name of student: _________________ (will be kept confidential)

Age in years: _______ Gender ☐ Female ☐ Male: Year of study: ☐ 3rd ☐ 4th ☐ 5th BMI: ___________
Family history of ☐ Diabetes ☐ Hypertension ☐ Hyperlipidemia ☐ cardiovascular ☐ others ________

Please answer the following questions as per your weekly routine regarding your dietary habits and lifestyle.

Section-I: Lifestyle and physical activity.

1. Do you exercise in your weekly routine? ☐ Yes ☐ No
2. If yes, what type of exercise do you perform? ☐ Aerobics ☐ walk ☐ Running ☐Yoga
3. How many days per week do you exercise? ☐ ≤ 3days ☐ > 3 days
4. Do you participate in sports in your weekly routine? ☐ Yes ☐ No
5. What type of sports do you participate in? ☐ Indoor ☐ Outdoor

Section-II: Dietary Habits.

1. Are you following a regular diet plan regularly? ☐ Yes ☐ No
2. Which meal of the day do you skip often? ☐ Breakfast ☐ Lunch ☐ Dinner ☐None
3. How often do you eat vegetables in a week? ☐ None/Occasionally ☐ ≤ 3days ☐ > 3 days
4. How often do you eat meat in a week? ☐ None/Occasionally ☐ ≤ 3days ☐ > 3 days
5. How often do you eat fruit in a week? ☐ None/Occasionally ☐ ≤ 3days ☐ > 3 days
6. How often do you eat dry fruits/nuts in a week? ☐ None/Occasionally ☐ ≤ 3days ☐ > 3 days
7. How often do you eat junk/fast food in a week? ☐ None/Occasionally ☐ ≤ 3days ☐ > 3 days
8. How often do you use energy drinks in a week? ☐ None/Occasionally ☐ ≤ 3days ☐ > 3 days
9. How often do you use carbonated drinks in a week? ☐ None/Occasionally ☐ ≤ 3days ☐ > 3 days

Section-III: Lipid Levels.

Volume of blood drawn _____ml

Total cholesterol _____mg/dl

HDL _____mg/dl

LDL _____mg/dl

Triglycerides _____mg/dl

Signature of student_____________________ Date and time: ____________________

Name and signature of recruiter____________________ Date and time: ____________________
